# Templating effect of single-layer graphene supported by an insulating substrate on the molecular orientation of lead phthalocyanine

**DOI:** 10.3762/bjnano.11.66

**Published:** 2020-05-19

**Authors:** K Priya Madhuri, Abhay A Sagade, Pralay K Santra, Neena S John

**Affiliations:** 1 Centre for Nano and Soft Matter Sciences, Jalahalli, Bengaluru 560 013, India; 2 Laboratory for Advanced Nanoelectronic Devices, Sir C. V. Raman Research Park, Department of Physics & Nanotechnology, SRM Institute of Science and Technology, Kattankulathur 603 203, Tamil Nadu, India

**Keywords:** conducting atomic force microscopy (C-AFM), lead phthalocyanine (PbPc), molecular orientation, single-layer graphene, substrate effect, two-dimensional grazing incidence X-ray diffraction (2D-GIXRD)

## Abstract

The influence of single-layer graphene on top of a SiO_2_/Si surface on the orientation of nonplanar lead phthalocyanine (PbPc) molecules is studied using two-dimensional grazing incidence X-ray diffraction. The studies indicate the formation of a mixture of polymorphs, i.e., monoclinic and triclinic forms of PbPc with face-on (lying down) and edge-on (standing up) PbPc orientations, respectively. The formation of monoclinic fractions is attributed to the presence of the graphene layer directing the π interactions between the highly delocalized macrocycles. The competing interfacial van der Waals forces and molecule–molecule interactions lead to the formation of a small fraction of triclinic moieties. The nanoscale electrical characterization of the thin PbPc layer on graphene by means of conducting atomic force microscopy shows enhanced vertical conductance with interconnected conducting domains consisting of ordered monoclinic crystallites through which the charge transfer occurs via tunneling. These results show the importance of a templating layer to induce the formation of a required phase of PbPc suitable for specific device applications.

## Introduction

**Figure 1:**
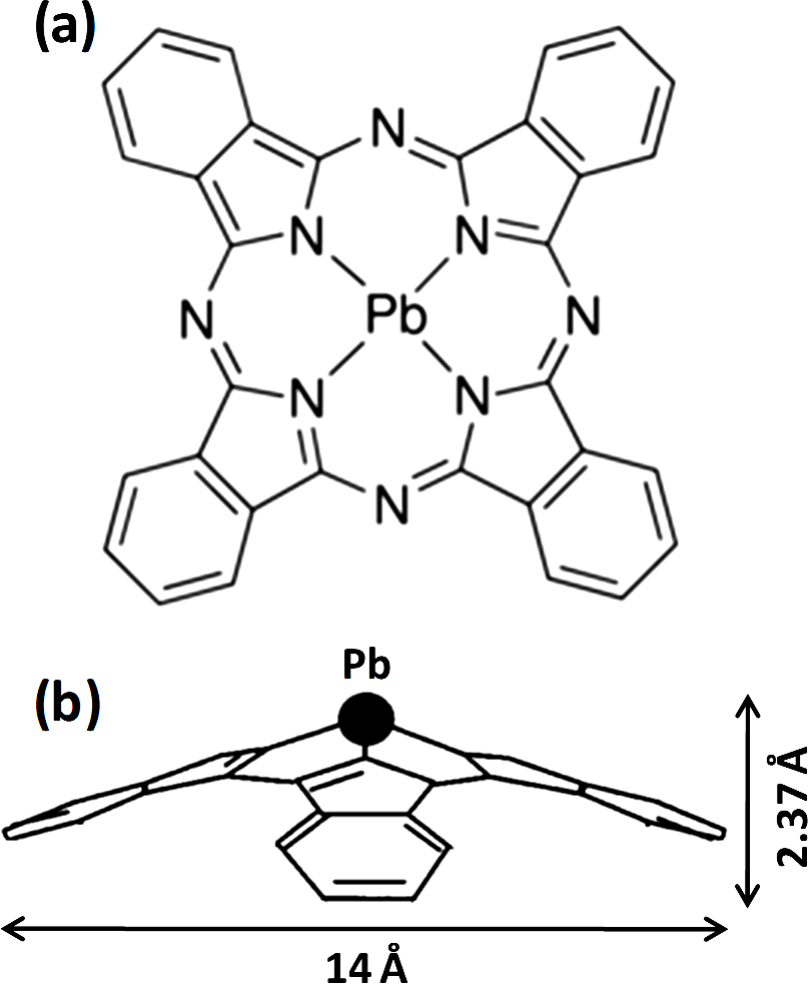
Chemical structure of lead phthalocyanine. (a) Top view and (b) side view of a Pb(II)Pc molecule.

Organic semiconductors have been extensively used in, among others, organic light-emitting diodes and organic photovoltaics. In particular, metal phthalocyanines (MPcs) have gained considerable interest as they offer flexibility in the modification of their optoelectronic properties through their molecular packing, which in turn is governed by substrate–molecule interactions [1–4]. Nonplanar MPcs, such as lead phthalocyanine (PbPc), are particularly interesting in the field of photovoltaics due to their extraordinary near-infrared (NIR) absorption. The chemical structure of a PbPc molecule is given in Figure 1. The well-known polymorphs of crystalline nonplanar MPcs are monoclinic and triclinic forms [5]. In thin films of MPcs, the molecules may attain face-on or edge-on orientations with respect to the substrate plane while forming the above crystal phases. The monoclinic fractions are known to have strong absorption in the visible range while the triclinic polymorph exhibits intense NIR absorption bands [3,6]. The formation of specific crystalline phases of nonplanar MPc molecules has been largely explored by introducing various substrate modifications or templating layers including 1*H*,1*H*,2*H*,2*H*-perfluorodecyltrichlorosilane (FDTS), MoO_3_ and CuI. A FDTS layer induces the edge-on arrangement regardless of the crystal phase, while a CuI interlayer induces the formation of triclinic PbPc moieties stacked face-on to the substrate yielding enhanced NIR absorption [6]. Organic molecules such as pentacene, fullerene and sexithiophene have also been utilized for inducing the growth of the triclinic phase [7–8]. These studies indicate that the growth of an organic film depends on the delicate interplay between the substrate–molecule and the molecule–molecule interactions. In the case of CuPc molecules deposited on C_60_ layers on highly oriented pyrolytic graphite (HOPG) or SiO_2_, it has been reported that CuPc attains different orientations resulting in substantial differences in donor–acceptor energy level alignment at the interface. Thus, ordering and orientation of these molecules significantly affect charge carrier injection and transport in semiconductor devices [7].

Pristine substrate surfaces of HOPG and Si themselves can induce orientation control over the growth of MPc structures without the aid of additional templating layers. In our earlier work, we have observed that orientation and molecular packing of nonplanar PbPc molecules are influenced by the nature of the substrate, which has been attributed to different substrate–molecule interactions [9]. With the application of 2D materials, such as graphene in device configurations, it is important to understand the orientation of MPc molecules on these atomically thin materials [10–11]. A single-layer of graphene can serve as a transparent conducting electrode and function as donor or acceptor when combined with suitable organic counterparts [12–13]. However, graphene itself is supported on a rigid substrate for device integration and, hence, it is important to consider any influence of the underlying substrate on the MPc orientation. It has been shown that a monolayer of graphene, due to its extremely thin nature, exhibits transparency to the wetting behavior on substrates where van der Waals forces are the dominant surface–water interactions [14]. The wetting transparency disappears with an increasing number of graphene layers, and the wettability approaches that of graphite. The underlying support substrate is reported to even influence the chemical reactivity of a monolayer of graphene [15].

Most of the orientation studies of MPc on graphene deal with planar MPc molecules such as FePc, CoPc, CuPc and ZnPc, which form layers in a face-on configuration [16–19]. A combination of brilliant synchrotron radiation and a highly sensitive 2D X-ray detector are employed to establish the structure and orientation of organic molecules such as MPcs, pentacene and P3HT [6,11,20–22]. Studies concerning the orientation of nonplanar MPcs on graphene are rare. Vanadyl phthalocyanine (VOPc) has been reported to attain edge-on configuration on a graphene surface [23]. The nucleation of CuPc on graphene is reported to be influenced by the underlying Ir(111) substrate [24]. It will be interesting to explore the molecular orientation of nonplanar PbPc on single-layer graphene supported on a substrate. In this study, we have investigated the molecular orientation of a PbPc film deposited on chemical vapor deposition (CVD)-grown graphene transferred onto a SiO_2_/Si substrate, using synchrotron two-dimensional grazing incidence X-ray diffraction (2D-GIXRD). We show that although graphene induces the face-on stacking of monoclinic domains, the underlying SiO_2_ substrate can still cause edge-on triclinic orientations as well. We also present the electrical current mapping of PbPc on graphene revealing interconnected highly conducting domains.

## Results and Discussion

**Figure 2:**
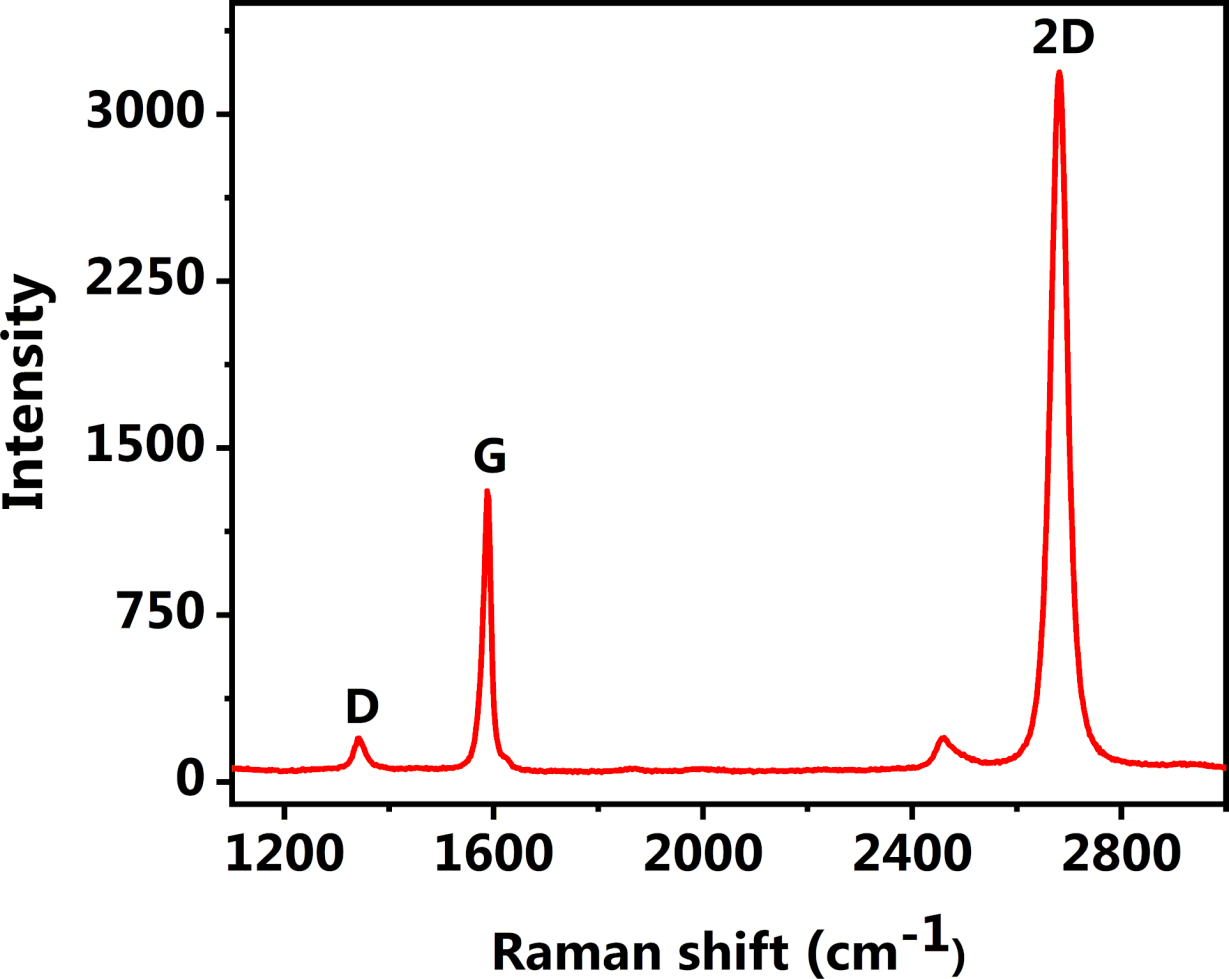
Raman spectrum of single-layer graphene on a SiO_2_/Si substrate used as a template for the deposition of the PbPc film.

The Raman spectrum of CVD-grown single-layer graphene transferred onto a SiO_2_/Si substrate (referred to as SLG/SiO_2_/Si hereafter) is presented in Figure 2. A sharp and strong peak at 2680 cm^−1^ corresponds to the characteristic 2D band of SLG and can be fitted with a single Lorentzian function (FWHM = 35.47 cm^−1^). The peaks at 1587 and 1342 cm^−1^ correspond to the G- and the D-band, respectively [25].

**Figure 3:**
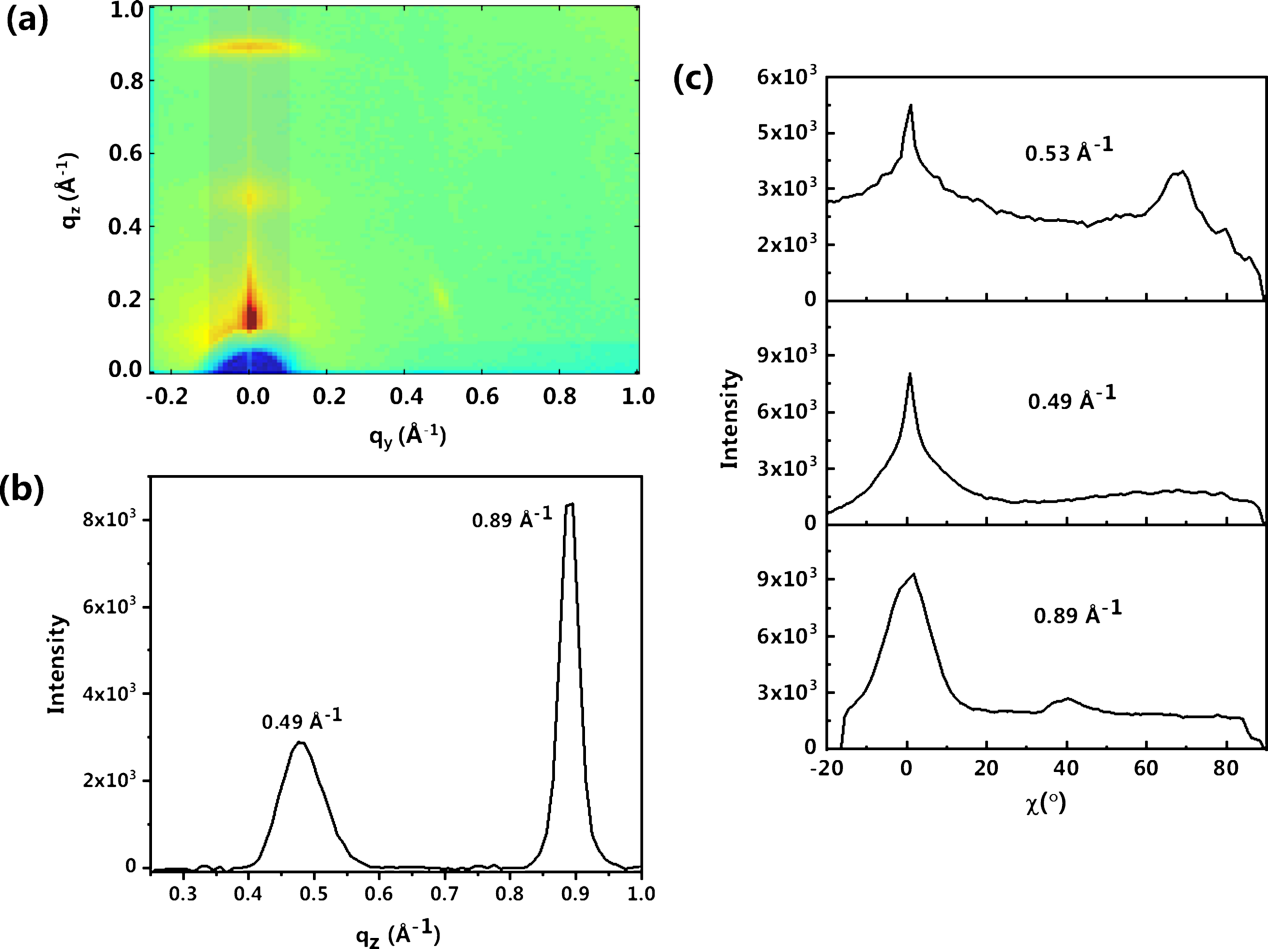
(a) 2D-GIXRD pattern of a 10 nm PbPc film on SLG/SiO_2_/Si. (b) Profile section along the *q*_z_ direction. (c) Intensity as a function of the azimuthal angle for different Bragg peaks.

The structure of the PbPc film on single-layer graphene was studied using 2D-GIXRD. Figure 3a shows the 2D-GIXRD pattern, which clearly shows a peak along the *q*_z_ direction at 0.89 Å^−1^, *d* = 7.06 Å, corresponding to the (320) reflection of monoclinic PbPc crystallites. Figure 3b shows the profile section of Figure 3a in *q*_z_ direction indicating the Braggs peaks as discussed above. The signature of monoclinic moieties along the *q*_z_ direction indicates that the PbPc molecules exhibit ordered stacking, normal to the substrate plane. This type of vertical ordering known as cofacial arrangement can promote charge transport in the vertical direction [6,9,26]. Further, a small fraction of the (320) reflection is measured at an azimuthal angle (Figure 3c). The 2D-XRD also shows the presence of a peak at 0.49 Å^−1^ along the *q*_z_ direction, which may be assigned to the (200) reflection of the monoclinic phase or the (001) reflection of the triclinic phase, occurring at the same positions with *d* = 12.82 Å (Figure 3a,b). This less intense peak indicates that there is a small number of crystallites arranged in an edge-on configuration [26]. There is a peak at 0.53 Å^−1^ corresponding to the (100) reflection of the triclinic phase in the *q*_xy_ plane with *d* = 11.87 Å. This signature in the *q*_xy_ plane may be an indication of a tilted face-on arrangement of triclinic moieties with respect to the substrate (Figure 3b,c). The average ratio between monoclinic and triclinic phase was roughly estimated from the corresponding Bragg peaks, and the monoclinic phase constitutes 65–70% of the PbPc crystallites.

The molecular orientation of both planar and nonplanar MPc molecules on oxide substrates such as SiO_2_ is well known. The molecules were shown to preferably have an edge-on orientation [3,7,9,27–29]. In our earlier studies, we have reproducibly obtained different crystallites of PbPc on substrates such as HOPG, Au(111), Si and SiO_2_. Detailed studies using Raman spectroscopy and 2D-GIXRD show ordered monoclinic and triclinic moieties on HOPG and Si substrates, respectively, while the Au(111) surface gives rise to disordered fractions due to the absence of long-range ordering [9,26]. In the present study, the presence of single-layer graphene on SiO_2_ has a templating effect and induces a monoclinic packing of PbPc in the face-on configuration. Our previous theoretical studies have shown that the PbPc molecule exclusively adopts a face-on configuration on an unsupported graphene layer owing to π–π and van der Waals interactions. For multilayer depositions, ordered π stacking of the macrocycles with Pb-up configuration is expected due to the dominant π–π interactions between the Pc macrocycle and the graphene layer [9,26].

**Figure 4:**
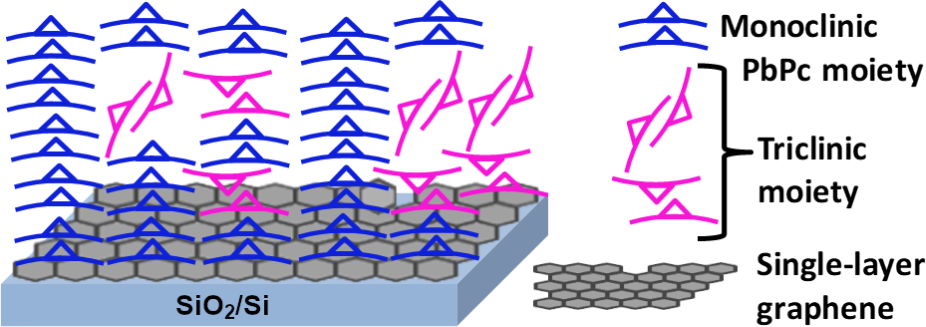
Schematic showing the molecular orientation of PbPc molecules on SLG/SiO_2_/Si.

In the present study, although there is a graphene layer, triclinic moieties with edge-on configuration are still observed. In the case of CVD graphene transferred on to substrates by the polymer method, it has been reported that the presence of polymer residues can cause edge-on orientation for pentacene on graphene [11]. However, in this work, effort has been taken to remove any polymer residues via thermal treatment [30]. Recently, Rafiee et al. found, in the context of wetting, that the van der Waals forces are not disrupted by the graphene sheet as it is extremely thin (ca. 0.3 nm) [14]. Hence, we consider that the edge-on configurations are formed under the influence of the underlying SiO_2_. The presence of triclinic moieties in an inclined configuration could be a result of competing interfacial van der Waals and forces and the π interactions between the graphene layer and the underlying SiO_2_. Thus more than one layer of graphene may be required to diminish the weak interfacial van der Waals forces from the SiO_2_ substrate [7]. The possibility of interaction between molecules in the top layers further away from the influence of graphene may also cause inclined and random orientations [31]. Based on the 2D-GIXRD measurements, a schematic of the molecular arrangement of PbPc molecules with monoclinic and triclinic fractions on the surface of SLG/SiO_2_/Si is inferred in Figure 4.

**Figure 5:**
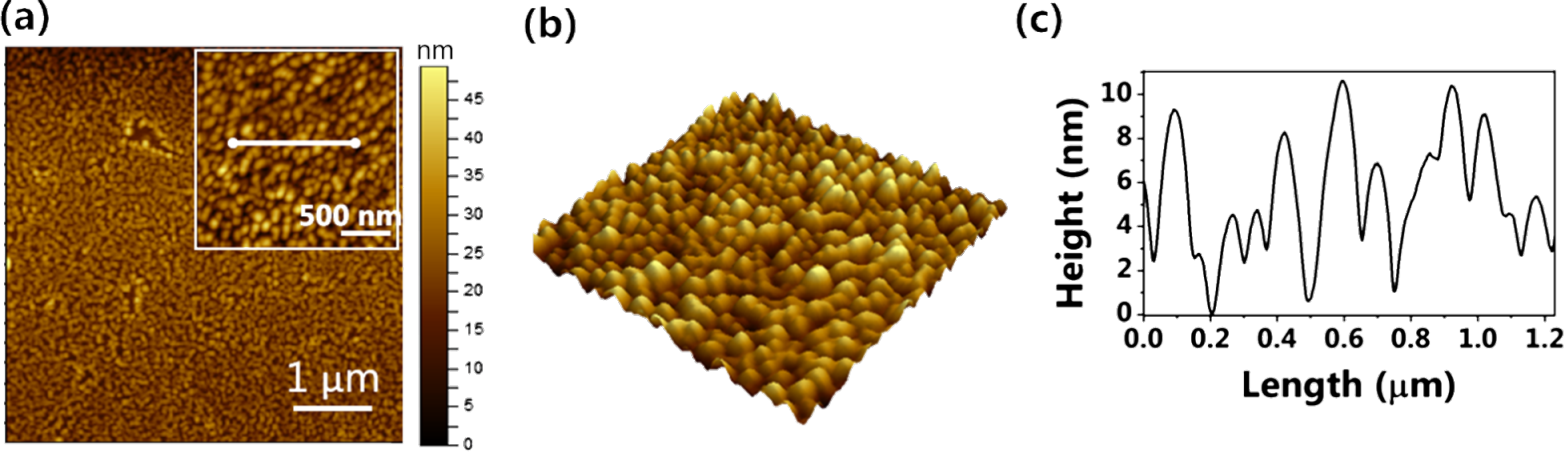
(a) AFM image of a 10 nm PbPc layer on single-layer graphene. The inset shows a magnified image of a 2 µm × 2 µm area. (b) 3D view of the inset image. (c) Profile section across the marked line in the inset of (a) showing the height variation.

The topography of the PbPc layer was studied using atomic force microscopy (AFM, Figure 5). Figure 5a and the inset show that the film consists of granular PbPc crystallites deposited uniformly on the surface of a single-layer graphene sheet. The size of the PbPc grains is 80–100 nm. The figures show that the film is quite continuous and uniform. Figure 5c shows the profile section taken from the inset of Figure 5a indicating the height variations across the film. The rms roughness of the film was found to be 2.82 nm. Wang et al. carried out a similar study by depositing a 10 nm thin ZnPc film on a graphene/SiO_2_/Si substrate to study the effects of the molecular orientation on the interfacial electronic properties. The roughness of the film was reported to be 2.47 ± 0.28 nm [19]. The crystallite size of PbPc was also derived from the 2D-GIXRD peaks at 0.49 and 0.89 Å^−1^ using the Scherrer equation with a value of *k* = 0.9 assuming spherical particles. Crystallite sizes of 14.37 and 33.58 nm were obtained from the Bragg peaks of 0.49 and 0.89 Å^−1^, respectively.

**Figure 6:**
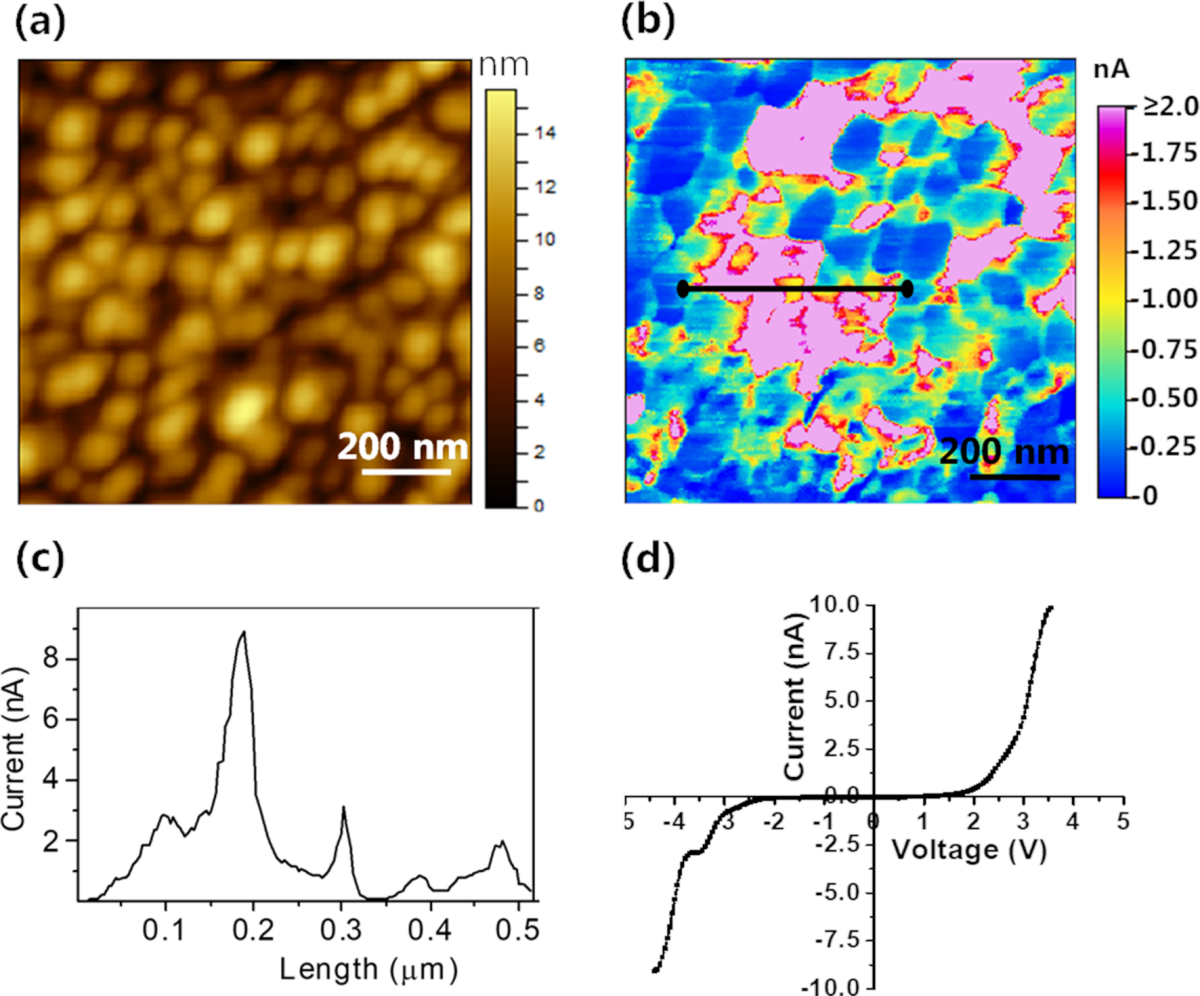
(a) AFM topography,1 µm × 1 µm scan area. (b) Corresponding current map of 10 nm PbPc thin film on SLG/SiO_2_/Si substrate obtained at 2 V sample bias. (c) Profile section of (b) along the marked line showing the current variation across the film. (d) *I*–*V* curve acquired from a conducting domain.

Further, we have carried out electrical studies using conducting-AFM (C-AFM). Figure 6a,b shows the topography and the corresponding current map of the film. The current response map shows an average current value of about 1 nA across the surface with highly conducting grains, which exhibit current values as high as 8–9 nA (Figure 6c). The conducting domains may arise as a result of face-on monoclinic fractions that are vertically stacked. Such stacked molecules can give rise to more energy states near the bandgap aiding charge transport [9]. The less conductive regions may have triclinic moieties or other crystallite arrangements that do not facilitate charge transport. Figure 6d is the *I*–*V* curve acquired from one of the conducting domains in the vertical configuration.

**Figure 7:**
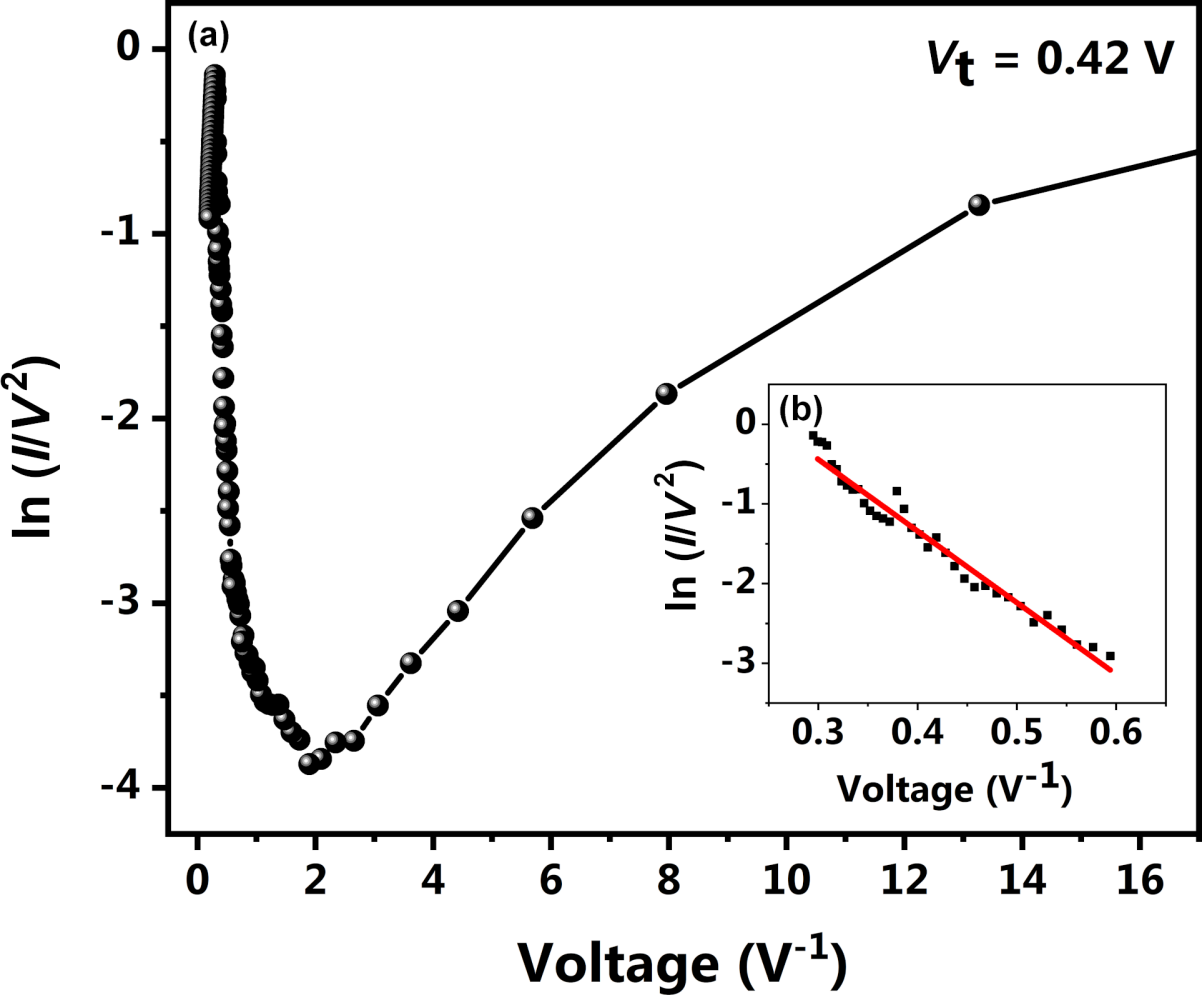
(a) Plot of ln(*I*/*V*^2^) as a function of *V*^−1^ for PbPc on single-layer graphene showing a transition from direct tunneling to F-N tunneling and (b) linear fit for the region of F-N tunneling.

The curve is non-linear as expected for PbPc and higher current values are observed. The obtained *I*–*V* plot can be fitted to a generalized Simmons equation indicating that the charge transport in a thin PbPc layer is governed by tunneling (Figure 7). A plot of ln(*I*/*V*^2^) as a function of *V*^−1^ indicates a logarithmic dependence in the low-bias region showing direct tunneling, which transforms into a linear dependence in the high-bias region, suggesting Fowler–Nordheim (F-N) tunneling or injection tunneling. However, it is seen that the transition from direct to F-N tunneling is not a sharp transition. Instead, there is a seemingly linear slope between the two states. A sharp rise in linear current is noticed beyond this region, which corresponds to F-N tunneling [9]. Graphene has similar properties as graphite and is expected to exhibit similar templating effects as observed earlier on graphite due to its sp^2^-hybridized structure. The vertically stacked monoclinic domains on the graphene surface provide an uninterrupted path for electrical transport and thus give rise to higher current values.

## Conclusion

Structural studies of a thin layer of PbPc (10 nm) deposited on CVD-grown single-layer graphene supported on a SiO_2_/Si substrate show the presence of a mixture of monoclinic and triclinic polymorphs with, respectively, face-on and edge-on configurations. The presence of monoclinic fractions in a preferred face-on orientation is ascribed to the π–π interactions between the sp^2^-hybridized plane of graphene and the macrocycles of PbPc. However, the presence of SiO_2_ surface beneath graphene, which exerts interfacial van der Waals interactions and intermolecular interactions on the top layers, influences the orientation of PbPc molecules leading to the formation of a very small fraction of triclinic moieties in edge-on or tilted configuration. Further, electrical characterization of these films in a vertical configuration shows enhanced conduction. Monoclinic domains stacked cofacially on the substrate surface facilitate the charge transport by improved π-electron coupling. These studies demonstrate the possibility to fabricate device architectures with the desired orientation of the film by carefully choosing the substrate or introducing a templating layer.

## Experimental

A 10 nm thin PbPc film on single-layer graphene supported on a SiO_2_/Si substrate (SLG/SiO_2_/Si) was deposited using physical vapor deposition. The PbPc film was deposited at a base pressure of 1 × 10^−5^ mbar while the substrate was held at 100 °C. The deposition rate was 1–1.5 Å·s^−1^. Single-layer graphene was synthesized by chemical vapor deposition (CVD) on a copper substrate and transferred by a standard technique using poly(methyl methacrylate) (PMMA) onto a SiO_2_ (300 nm)/Si substrate as reported elsewhere [30]. This process is optimized with regard to minimal PMMA contamination. Further after the transfer, the substrates were heated to 400 °C in forming gas for 1 h to remove remaining polymer residues.

The structural characteristics of the single-layer graphene were studied using a Horiba XploRA PLUS Raman spectrophotometer with a 532 nm laser and a 50× objective. The molecular orientation of the thermally deposited PbPc film on single-layer graphene was studied using 2D grazing incidence X-ray diffraction (2D-GIXRD) at the PETRA III P08 beamline (Deutsches Elektronen-Synchrotron, DESY) with a flat-panel Perkin Elmer detector with 2048 × 2048 pixels of the size of 200 µm at a beam energy of 25 keV, corresponding to λ = 0.496 Å. The obtained results were analyzed with the help of the GIXSGUI software with MATLAB interface [32].

Morphological studies and electrical measurements of the PbPc layer were carried out using an Agilent 5500 AFM. The AFM was operated in C-AFM mode in which the PbPc layer is sandwiched between the conductive graphene layer, which served as a bottom electrode, and the Cr/Au tip (diameter < 35 nm and *k* = 0.18 N·m^−1^, MikroMasch, USA), serving as a top electrode.
